# The cost-effectiveness of oral contraceptives compared to ‘no hormonal treatment’ for endometriosis-related pain: An economic evaluation

**DOI:** 10.1371/journal.pone.0210089

**Published:** 2019-01-30

**Authors:** Tobias Sydendal Grand, Hasan Basarir, Louise J. Jackson

**Affiliations:** 1 Health Economics Unit, University of Birmingham, Birmingham, United Kingdom; 2 RTI Health Solutions, Greater Manchester, United Kingdom; Universitat Bremen, GERMANY

## Abstract

**Objective:**

To develop a preliminary cost-effectiveness model that compares oral contraceptives and ‘no hormonal treatment’ for the treatment of endometriosis-related pain.

**Methods:**

A *de novo* preliminary state transition (Markov) model was developed. The model was informed by systematic literature review and expert opinion. The uncertainty around the results was assessed both by deterministic and probabilistic sensitivity analyses. The economic evaluation was conducted from National Health Service (NHS) England perspective. The main outcome measure was incremental cost per quality-adjusted life year (QALY), with cost-effectiveness plane and cost-effectiveness acceptability curves presented for alternative willingness-to-pay thresholds.

**Results:**

Oral contraceptives dominated ‘no hormonal treatment’ and provided more QALYs at a lower cost than ‘no hormonal treatment’, with a cost-effectiveness probability of 98%. A one-way sensitivity analysis excluding general practitioner consultations showed that oral contraceptives were still cost-effective.

**Conclusions:**

The analyses showed that oral contraceptives could be an effective option for the treatment of endometriosis, as this treatment was shown to provide a higher level of QALYs at a lower cost, compared to ‘no hormonal treatment’. The results are subject to considerable parameter uncertainty as a range of assumptions were required as part of the modelling process.

## Introduction

Endometriosis is defined as the presence of endometriosis-like tissue outside the uterus, which induces a chronic, inflammatory reaction. It is an oestrogen-dependent condition with a prevalence rate of 4–10% in reproductive age women, which decreases to 2–5% after menopause[1]. Symptoms such as severe dysmenorrhea, deep dyspareunia, chronic pelvic pain, ovulation pain, cyclical or perimenstrual symptoms with or without abnormal bleeding, infertility and chronic fatigue have been observed in patients with endometriosis[2]. There is no established cure for endometriosis. The economic burden of endometriosis is considerable and indicated to be similar to other chronic diseases, such as diabetes, Crohn’s disease and rheumatoid arthritis, due to productivity losses, delayed diagnosis and comorbidities [3]. The definitive method to diagnose endometriosis is by laparoscopic surgery, but due to the invasive nature of such surgery, medical therapy such as oral contraceptives (OCs) is recommended for women presenting with symptoms suggestive of endometriosis[4, 5].

Current guidelines on hormonal therapies such as OCs to treat endometriosis-related pain are based on limited clinical evidence, and in particular, earlier studies often failed to include placebo or no treatment as comparator[5]. Recent guidance by the National Institute for Health and Care Excellence (NICE) has stated that more research is needed to evaluate whether pain management programmes are a clinically and cost-effective intervention for women with endometriosis[6]. The research undertaken in this economic evaluation outlines the development of an appropriate model structure, which was used to investigate the cost-effectiveness of oral contraceptives for endometriosis-related pain compared to ‘no hormonal treatment’ (treatment with analgesics only). The research thus aimed to assess whether the existing guidelines on the treatment of endometriosis with oral contraceptives (in the absence of a confirmed diagnosis) is likely to be cost-effective, based on the current evidence available.

### Objective

To develop preliminary cost-effectiveness model that compares OCs versus ‘no hormonal treatment’ for endometriosis-related pain.

## Methods

A decision analytic model informed by a systematic literature review and expert elicitation was built to assess the cost-effectiveness of OCs to treat endometriosis-related pain.

### Systematic review

Scoping searches in Medline and Embase were conducted between the 1^st^ to 10^th^ of June 2016 and are listed in S1–S4 Tables and were undertaken to develop the eligibility criteria and literature searches. An adapted population, intervention, comparator, outcomes (PICO) framework was used to structure the search and eligibility criteria. S5 Table lists the inclusion and exclusion criteria using the PICO framework. The eligibility criteria were aligned with current recommendations from NICE, the Cochrane handbook for systematic reviews of interventions, and guidance from the Centre for Review and Dissemination[7–9]. Five electronic databases, Medline, Embase, NHS EED, HTA and DARE were searched between the 13^th^ to 29^th^ of June 2016 (S6–S8 Tables). The OVID platform was used to construct two separate searches for Medline and Embase, whereas NHS EED, HTA and DARE that were accessed from the Centre for Review and Dissemination database were searched using keywords, such as endometriosis and pain. The search terms were keywords and medical subject headings were retrieved from the thesaurus of the individual databases, to avoid inconsistency in MeSH definitions[10]. A hand-search was also conducted to identify relevant publications that may have not been sourced by the electronic database (S9 Table).

## Categorisation

The studies were categorised over two stages following a process outlined by Roberts and colleagues[11]. The first stage involved screening titles and abstracts, and assessing the studies for inclusion against eligibility criteria and categorisation. The second stage involved a screening of the full papers, assessment for inclusion and further categorisation. The two-stage process and the categorisation used are shown in S10 Table.

## Quality assessment and data extraction

Economic evaluations were assessed by a checklist for economic evaluations[12]. Their results were extracted and compared. Studies that were not classified as full economic evaluations were assessed by relevant parts of the checklist to allow for a quality assessment. The purpose of quality assessment was to assess the quality of papers that included input parameters, which could inform the decision analytic modelling (DAM) and to prioritise the most appropriate parameters for the modelling stage.

### Decision analytic model

**Fig 1:**
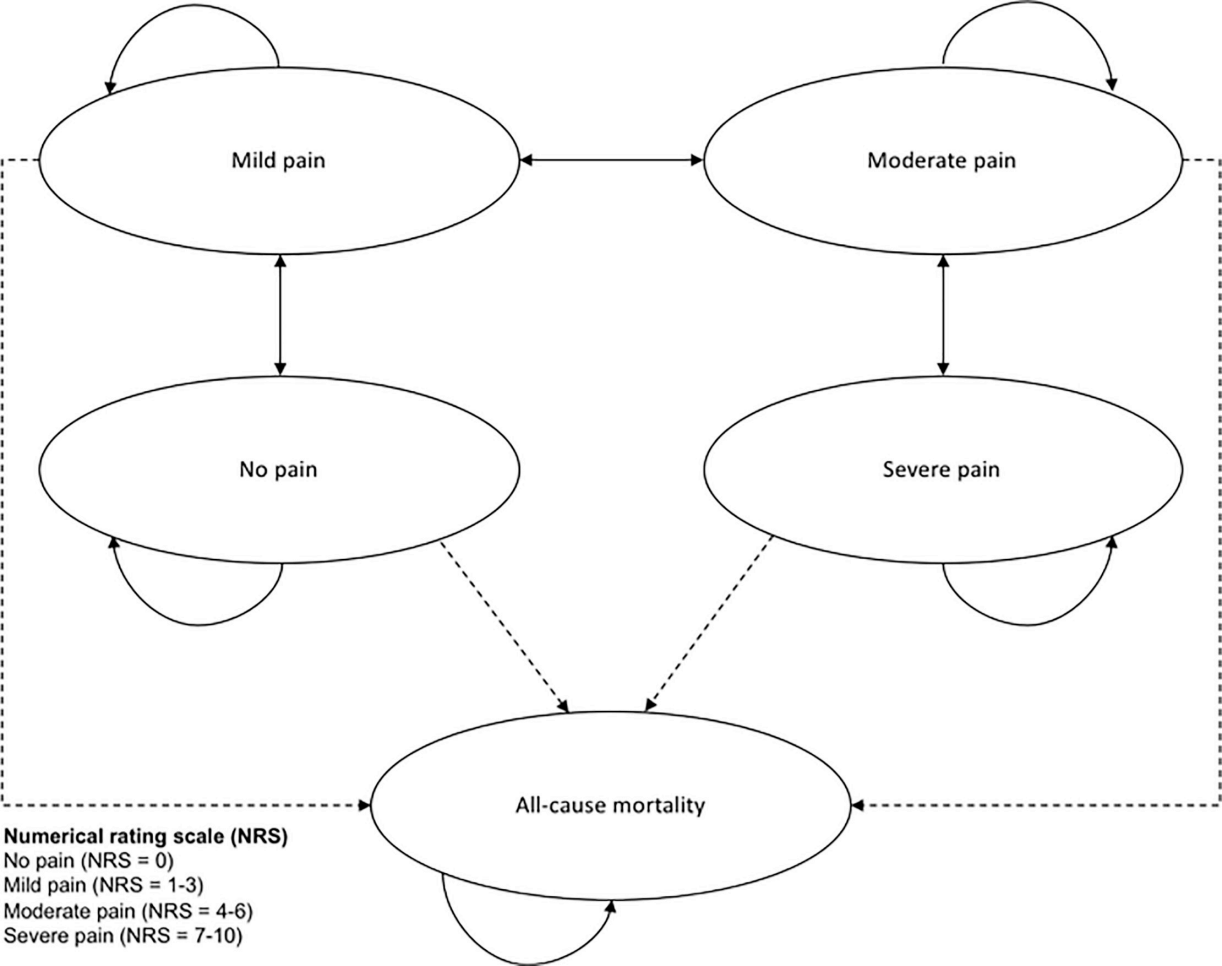
Model structure. Markov transition state model for endometriosis-related pain.

A preliminary Markov state transition model, informed by systematic literature searches and expert opinion, was constructed in MS Excel. Given the limited evidence on endometriosis-related pain, this model provided an initial framework of undertaking an economic evaluation on this condition, for which the recommended initial treatment is pain management. The intervention arm was OCs as this is the first line treatment in the UK [5]. The control arm was ‘no hormonal treatment’, with treatment with pain relief only, due to uncertainty and delay of diagnosis. The starting age of the cohort was 32 years, which has been estimated as the mean age at diagnosis[13]. The model was run until the mean age at menopause, which has been estimated as 50 years[1, 14, 15]. A cycle length of one month was considered appropriate, as it would allow changes in symptoms and treatments to be captured. Half cycle corrections were applied following recommendations by Philips et al. [16]. The analysis took a National Health Service (NHS) England perspective and assessed incremental differences in cost and quality-adjusted life years (QALY), which were discounted by 3.5% as recommended by NICE[17]. The Markov model had five health states, namely ‘no pain’, ‘mild pain’, ‘moderate pain’, ‘severe pain’ and ‘all-cause mortality’. Each health state was defined by the numerical rating scale (NRS) for pain in accordance with Breivik et al. [18], where no pain corresponds to a NRS value of zero, mild pain to values from one to three, moderate pain to values four to six and severe pain to values from seven to ten. The model structure is shown in Fig 1, where transition states of the model are represented by the ovals, and transitions between states are represented by the transition arrows. The transition arrows leading to all-cause mortality were dotted to illustrate that endometriosis does not contribute to mortality, but that all-cause mortality was included.

## Cost parameters

The cost parameters were informed by the British National Formulary and Personal Social Services Research Unit publications (Table 1)[19, 20]. The use of analgesics in the treatment of endometriosis is common. It was assumed that individuals in this model would increase their use in correspondence with their pain intensity in both arms[21]. The resource use of ibuprofen and paracetamol was gradually increased from the mild pain state to the severe pain state, as the severity of pain scores increased based on the NRS. The dosing followed recommendations from The Royal Pharmaceutical Society[19]. In line with these recommendations, the analgesics received by patients for the health states were assumed to be: for the mild pain state, half of the maximum dose of paracetamol; for the moderate pain state, the maximum dose of paracetamol; and for the severe pain state, the maximum dose of paracetamol and ibuprofen. The maximum dose of paracetamol and ibuprofen were 1000mg four times a day and 400mg three times a day, respectively. In line with current evidence, it was assumed that the frequency of general practitioner (GP) consultations would be every third month for the patients with ‘no hormonal treatment’ due to difficulties in diagnosis, in comparison to every 6 months for patients receiving OCs[5, 13].

**Table 1:** Cost data.

	Unit cost (£)	Source	Distributions
**Medical therapy**			
Combined oral contraceptive (microgynon)	2.82	[19]	Gamma
Consultation every 6^th^ month (GP^a^ 10 min)	26.67	[20]	Gamma
**No hormonal treatment**			
Consultation every 3^rd^ month (GP 10 min)	26.67	[20]	Gamma
**Usual care per cycle^b^**			
Ibuprofen (Ibucalm)	2.43	[19]	Fixed
Paracetamol (Mandanol)	2.31	[19]	Fixed

An overview of the resource use, sources and assigned distributions used in the economic evaluation.

^a^General practitioner.

^b^Costs assigned to both ‘no hormonal treatment’ and oral contraceptives.

## Utility parameters

Two studies were identified which contained quality of life weights for endometriosis-related pain. A study by Simoens et al. (3) estimated endometriosis-related symptoms, where endometriosis-related pain was indicated as the main reason for the reduction in quality of life, but the study did not consider different severity levels of pain. In contrast, a study concerned with dysmenorrhea, reported SF-36 scores for different severity levels of pain, which were converted to QALYs for each health state, but dysmenorrhea represents only one of several types of pain related to endometriosis[22, 23]. None of the estimates from these studies could be used directly as they related to different health states or did not include all aspects of endometriosis-related pain. To obtain utility estimates that were relevant to the model developed, expert elicitation was employed. A gynaecologist was presented with the available evidence and provided estimated utility values for each health state using a web-based online elicitation tool called MATCH Uncertainty Elicitation Tool^24^. The method chosen for the MATCH Uncertainty Elicitation Tool followed the roulette or “chips and bins” method[24, 25]. The utility values are shown in Table 2 and were adjusted to the time horizon of one month in the cost-effectiveness analysis.

**Table 2:** QALY data.

Health state	Data source[3] (QALY)	Data source[23] (QALY)^a^	Gynaecologist Roulette method (QALY)	PSA distribution^b^	Distribution
No pain	N/A	0.767	0.905	(27.357, 3.153)	Beta
Mild pain	0.809 Standard deviation 0.193	0.754	0.802	(82.023, 18.242)	Beta
Moderate pain		0.712	0.718	(55.931, 22.142)	Beta
Severe pain		0.686	0.573	(19.336, 14.487)	Beta

Sources of QALY data and the values obtained by the Roulette method used in the economic evaluation.

^a^SF-36 for dysmenorrhea converted to QALYs

^b^Probabilistic sensitivity analysis, alpha and beta values. PSA distributions and QALYs are rounded to three decimals.

## Transition probabilities

In the absence of appropriate evidence from the systematic literature review, all transition probabilities were informed by expert opinion. To elicit expert opinion, 1000 hypothetical patients were assigned to each health state and the rates for monthly transitions to the other health states were estimated by an expert in the field of gynaecology. Beta distributions for binomial and Dirichlet distributions for multinomial health states were fitted to the number of monthly transitions using common practice for health economic modelling[26]. The transition probabilities used in the model are shown in Table 3.

**Table 3:** Transition probabilities.

Transition probabilities	No hormonal treatment	PSA^a^	Oral contraceptives	PSA	Distributions
No pain to mild pain	0.003	(3, 997)	0.001	(1, 999)	Beta
Mild pain to no pain	0.002	(2, 998)	0.003	(3, 997)	Dirichlet
Mild pain to moderate pain	0.002	(2, 998)	0.0015	(1.5, 998.5)	Dirichlet
Moderate pain to mild pain	0.001	(1, 999)	0.003	(3, 997)	Dirichlet
Moderate pain to severe pain	0.003	(3, 997)	0.0001	(0.1, 999.9)	Dirichlet
Severe pain to moderate pain	0.0001	(0.1, 999.9)	0.004	(4, 996)	Beta

Transition probability parameters used in the economic evaluation.

^a^Probabilistic sensitivity analysis, alpha and beta values.

## Model assumptions

A number of assumptions were made to inform the modelling process, based on the literature and clinical inputs.

- It was assumed, based on clinical opinion, that moving across more than one health state within the cycle length of one month would not occur. For example, moving from the health state mild pain, to the health state severe pain within the same month was not permitted in the model.
- The impact of OCs on infertility was not considered in the model, as clinicians are not recommended to prescribe hormonal treatment for suppression of ovarian function to improve fertility[5].
- It was assumed that endometriosis does not contribute to all-cause mortality, which reflects the current evidence[4].
- The impact of side effects on cost and health-related quality of life related to OCs was considered to be negligible, as OCs are associated with long term safety, and was not included in the model[5].
- Surgery was not considered as part of the model, as medical therapy before and after surgery, is distinct from medical therapy to treat endometriosis-related pain[5].
- Outcomes other than endometriosis-related pain, such as abnormal bleeding or chronic fatigue, were not considered, because the focus of the study was on endometriosis-related pain.

## Sensitivity analyses

To assess uncertainty of the model input parameters, a one-way sensitivity analysis was conducted on costs. GP consultations were excluded in the one-way sensitivity analysis, as the frequency used in the base-case analysis was partly based on expert advice and were expected to have an impact given that GP consultations represent higher cost relative to the medication cost included in the analysis. Probabilistic sensitivity analysis (PSA) was conducted to assess the parameter uncertainty around the input parameters simultaneously. This involved 1000 random samples being drawn from the distributions assigned for the PSA, which were illustrated by a cost-effectiveness plane. A cost-effectiveness acceptability curve (CEAC), which showed the probability of cost-effectiveness at a range of willingness-to-pay thresholds, was also reported.

## Results

### Systematic review

**Fig 2:**
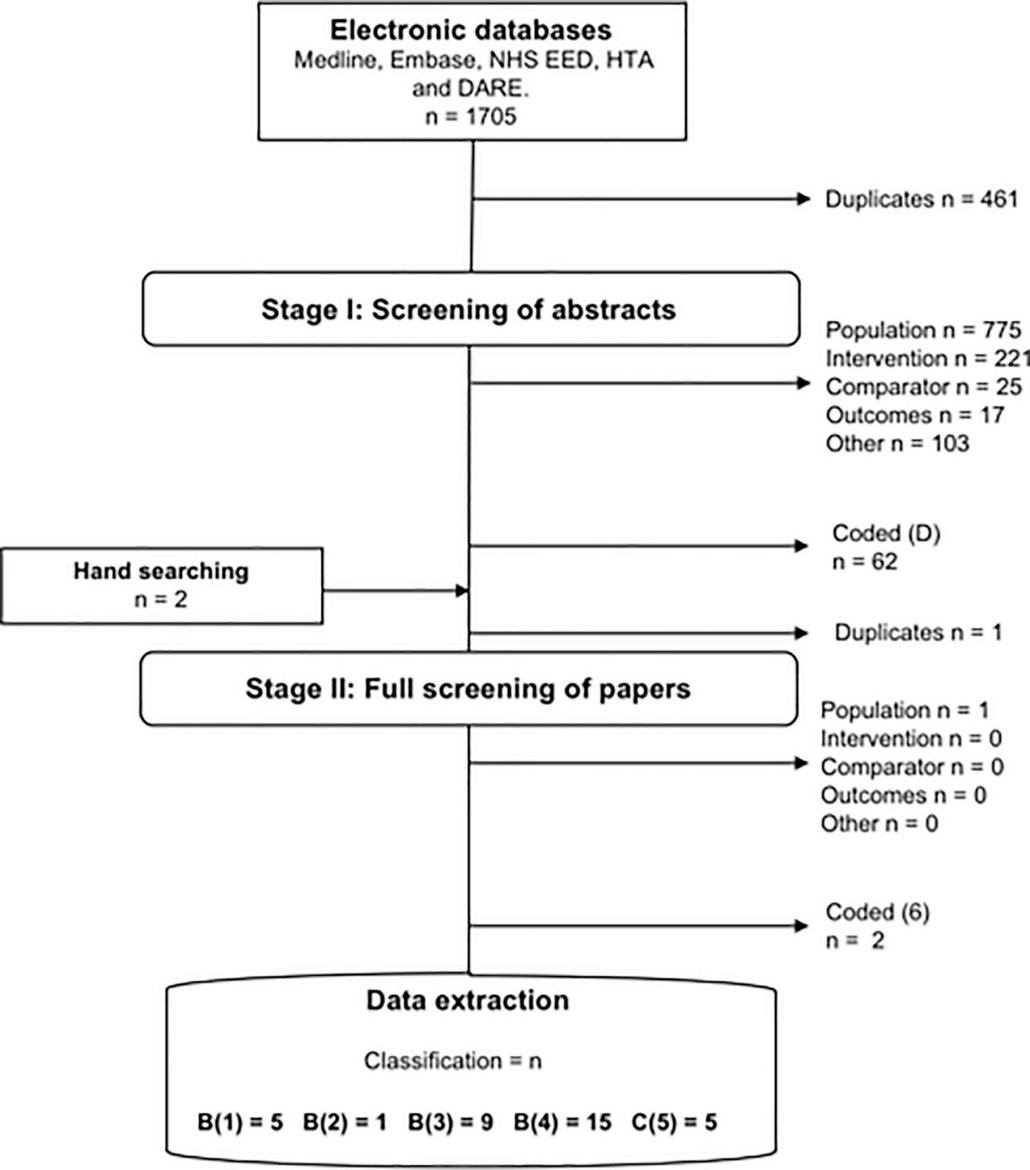
PRISMA diagram. Overview of the data extraction process.

The electronic database search identified 1705 published papers, of which 461 were duplicates. 35 papers were included for data extraction after full screening against the inclusion and exclusion criteria. In S11–S14 Tables, the 35 papers identified are presented, 6 papers were economic evaluations, 9 were cost studies, 15 were utility studies and 5 papers were classified as other relevant studies. Fig 2 illustrates the initial exclusion and classification of papers after screening the abstracts, followed by subsequent exclusion and classification after full screening.

## Economic evaluations

Overall, six economic evaluations were identified [27–32] and five of these were conference abstracts or posters. Health service perspectives were included in all economic evaluations. Five economic evaluations were cost-utility analyses reporting cost per QALY and one economic evaluation was a cost-effectiveness analysis reporting time until reduction of symptoms and costs. One economic evaluation was a full publication and the others were abstracts or poster presentations. All economic evaluations were concerned with endometriosis-related pain, except the study by Sanghera et al. [27] that was concerned with treatments to prevent recurrence of endometriosis following surgery. The comparators in the economic evaluations for endometriosis-related pain were OCs containing progestogen only or in combination with oestrogen, goserelin and leuprolide acetate, and self-care. For treatments to prevent recurrence of endometriosis following surgery the comparators were levonorgestrel-releasing intrauterine system, depot-medroxyprogesterone acetate and no treatment. Many studies failed to describe the details of the model structures, but Arakawa et al. (29) described a Markov model that classified endometriosis into two transition states by severity, a transition state for dysmenorrhea and all-cause mortality. Simple sub-medical states adherent to the transition states were included to reflect patient pathways. A study by Marmarali et al. (28) used a Markov model with three transition states to reflect endometriosis-related symptoms, treatment response, unresponsive to treatment and death. These two economic evaluations were the only analyses for endometriosis-related pain with a description of the model structures used.

### Decision analytic model

The outcome of the base-case analysis are provided in Table 4. The decision analytic model produced an estimate of cost for ‘no hormonal treatment’ of £1,707 and 9.88 QALYs gained, whereas OCs were £1,113 and 10.31 QALYs gained. The cost associated with ‘no hormonal treatment’ was therefore £594 more and provided 0.43 less QALYs than OCs. OCs therefore dominated ‘no hormonal treatment’, which means that the base-case interpretation was that OCs should be recommended over ‘no hormonal treatment’ at any given willingness-to-pay threshold.

**Table 4:** Base-case analysis.

Summary of base case deterministic results	Total costs (£)	Total QALYs^a^	ICER^b^
No hormonal treatment	1707	9.88	Dominated
Oral contraceptives	1113	10.31	
Mean difference	-594	0.43	

Summary of base-case deterministic results.

^a^QALYs are rounded to two decimals.

^b^Incremental cost-effectiveness ratio.

## One-way sensitivity analysis

The results of the one-way sensitivity analysis are shown in Table 5. The exclusion of GP consultations showed that ‘no hormonal treatment’ represented lower costs than OCs, which was in contrast to the base-case analysis, and a mean cost difference of £4 between ‘no hormonal treatment’ and OCs, which was £-594 in the base-case analysis. This one-way sensitivity analysis demonstrated that OCs were cost-effective at a threshold of £9 per QALY, if GP consultations were removed from consideration.

**Table 5:** One-way sensitivity analysis.

One-way sensitivity analysis	Total costs per intervention (£)	Total QALYs per intervention^a^	ICER^b^
No hormonal treatment	604	9.88	9
Oral contraceptives	608	10.31	
Mean difference	4	0.43	

^a^QALYs are rounded to two decimals.

^b^Incremental cost-effectiveness ratio.

## Probabilistic sensitivity analysis

**Fig 3:**
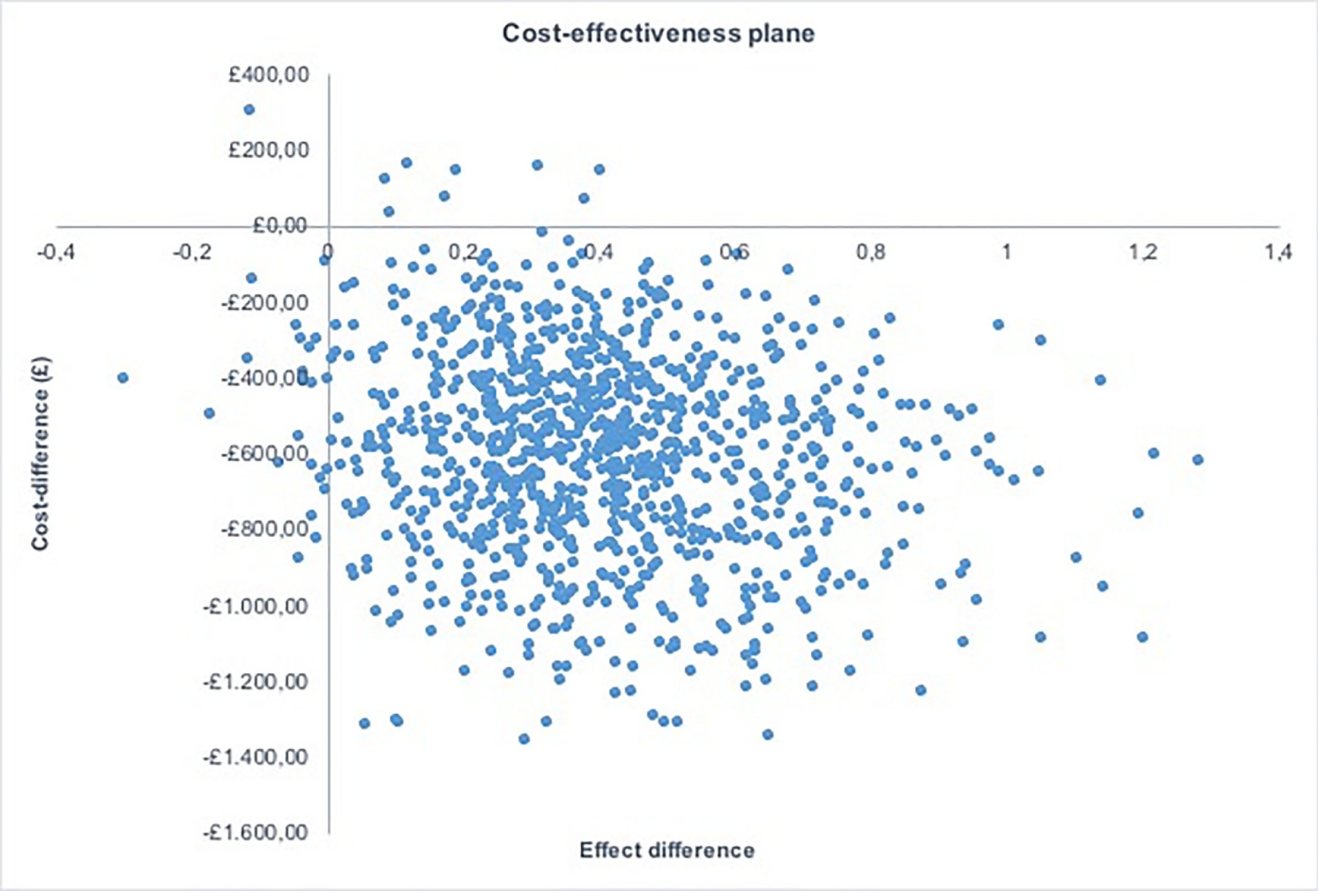
Cost-effectiveness plane. Showing the iterations from the probabilistic sensitivity analysis with cost difference along the y-axis and effect difference along the x-axis.

Fig 3 shows the results of PSA by illustrating the cost difference and effect difference for each sample drawn. Overall, 98% of the simulations were located in the South East quadrant of the cost-effectiveness plane, which demonstrated that ‘no hormonal treatment’ is highly likely to be dominated by OCs under a range of plausible variations in parameter estimates.

**Fig 4:**
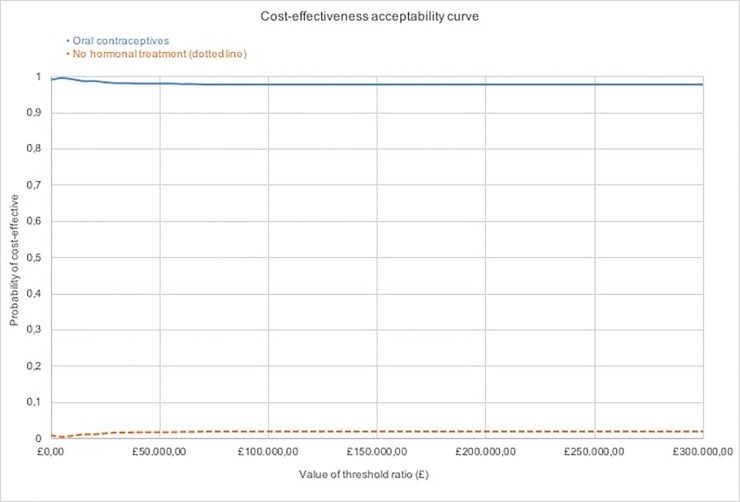
Cost-effectiveness acceptability curves. Showing the probability of cost-effectiveness along the y-axis and the value of threshold ratio along the x-axis.

The CEAC in Fig 4 shows that OCs have a higher probability of being cost-effective at any given threshold. The probability of ‘no hormonal treatment’ being cost-effective peaks at 0.02 probability of being cost-effective at thresholds above £50,000. OCs are recommended at any given willingness to pay threshold.

## Discussion

### Main findings

The literature review highlighted that there are very few economic evaluations conducted in this clinical area. The model-based cost-effectiveness analysis showed that OCs dominated ‘no hormonal treatment’, as OCs are less costly and provide more QALYs than ‘no hormonal treatment’, with a probability of 98% of being cost-effective at the accepted NICE threshold. The higher costs associated with ‘no hormonal treatment’ can be explained by the higher frequency of GP consultations, which was every third month for ‘no hormonal treatment’ in comparison to every 6 months for the OCs. The one-way sensitivity analysis showed that OCs were cost-effective at a threshold of £9 per QALY. The PSA showed that OCs were cost-effective at any given threshold. Therefore, the cost-effectiveness analysis suggests that OCs are cost-effective compared to ‘no hormonal treatment’.

### Strengths and limitations

This cost-effectiveness analysis has an exclusive focus on OCs to treat endometriosis-related pain, which has been recognised as an area where research is needed[5, 6]. To our knowledge, this is the first cost-effectiveness model structure concerned with endometriosis-related pain that was informed by a pain scale. The strength of this model is the ability to capture pain related to endometriosis exclusively, as different types of pain can have different durations and intensities. Previous economic evaluations have tended to classify endometriosis by severity stage which does not reflect the severity of endometriosis-related pain or predict the likely response to medical therapy[4, 33]. This is an important distinction, as for endometriosis, medical therapies are prescribed in accordance with pain rather than classification by severity stage[34, 35]. In this economic study the health states in the model were defined by endometriosis-related pain in the form of the numerical rating scale rather than classification by disease severity. The model has the potential to inform future research in this area, as it is the first one to provide the flexibility to incorporate the impact of medical therapies on different levels of endometriosis-related pain.

Nonetheless, there were some weaknesses associated with this study. The evidence to inform the input parameters of the DAM was limited. Due to the scope of the study, not all medical therapies that could potentially be used to treat endometriosis-related pain were included in this analysis, which is a limitation, but the most appropriate comparator (no hormonal treatment) was included.

The limited available evidence on modelling approaches meant that a model structure could not be developed based on previous work, and that external validity could not be explored through simulation and comparison to other sources such as clinical trials. This conclusion was shared by Sanghera et al. (27) who produced a model concerned with recurrence of endometriosis after surgery, and highlighted that the evidence available to inform the DAM was extremely limited. Face validity was established together with a clinical expert to ensure that the model components reflected endometriosis-related pain, and cross validation (comparison with other models) was undertaken but this was limited by the low number of cost-effectiveness studies concerned with endometriosis.

Although a number of assumptions were made, the model can be expanded by relaxing these assumptions as the evidence becomes available over time[5]. For example, it would be possible to develop the model to include treatment by surgery if additional data on cost and quality of life were available[36]. Endometriosis-related pain studies are often limited in sample size and duration, which meant that the impacts of side-effects and the discontinuation of treatment could not be considered as part of the economic evaluation[5].

### Implications

This study has implications for decision makers. The model supports current guidelines which suggest that treatment with hormonal treatment should be initiated on the basis of symptoms, rather than on a confirmed diagnosis of endometriosis. The model also has the potential to inform further decision making in this area. For example, the model could also be used to explore the benefits of early initiation of treatment for endometriosis. The starting age of the cohort in this study was informed by the current average age at diagnosis, but the age of initiation could be varied to explore the benefits of early treatment with OCs to inform the development of future guidelines. The current expert recommendation is to consider a diagnosis of endometriosis in the presence of symptoms such as dysmenorrhea, non-cyclic pelvic pain and others. However, the evidence on symptoms that indicate a diagnosis of endometriosis is weak and incomplete, and hence there could be a case for starting women on hormonal treatment earlier than currently occurs[5].

There are also implications for future research. This study adds valuable evidence in an under-researched area and demonstrates the challenges of decision analytical modelling for endometriosis-related conditions.

A number of assumptions needed to be included in this analysis to reflect the complexity of endometriosis-related pain. There is a lack of data in this area in relation to health state utility values and disease progression. Without such clinical data to inform economic evaluations for endometriosis-related pain, expert opinion provides an alternative source of information. Extensive sensitivity analyses were included in this study to explore the uncertainty associated with such estimates. Sanghera et al. (27) also used elicitation methods to derive expert opinion from two trial clinicians for both utilities and transition probabilities using similar methods to those used in this study and also highlighted the lack of evidence in this area. In this analysis, only one clinical expert was consulted for expert opinion due to resource constraints. Several other economic evaluations identified in the literature searches used expert opinion to inform their analysis, but included little or no information on the methods used to derive the point estimates, and on the extent that the expert opinions had informed the research [29–31].

In this analysis, initiation with OCs were considered exclusively in regards to endometriosis-related pain, but this treatment can also be initiated for other reasons and for example discontinued should a woman want to conceive. Many factors can influence treatment, but it was necessary in respect to the study to assume that initiation of OCs was focused on treating endometriosis-related pain. OCs are reported to have less side-effects than hormonal alternatives such as gonadotropin-releasing hormone, and with the great advantage that they can be taken indefinitely [37]. Tolerability and side-effects can vary significantly with the treatment, and further work is needed in this area.

In regards to pharmaceutical costs, it is probable that women would use other pain killers more efficacious than the ones included in this analysis, such as codeine or tramadol. However, these pain killers also do not represent significant costs, and it was decided to focus on ibuprofen and paracetamol. More research has the potential to limit the number of assumptions necessary for economic evaluations. Rigorously testing these assumptions by transparent sensitivity analyses can provide confidence in the results in the absence of primary data.

## Conclusion

The results of the preliminary cost-effectiveness analyses conducted to compare OCs with ‘no hormonal treatment’ in the treatment of endometriosis-related pain showed that OCs are less costly and more effective on average. There was considerable uncertainty around some of the input parameters, as these were based on expert opinions and assumptions. However, extensive sensitivity analyses demonstrated that OCs are highly likely to be cost-effective compared to ‘no hormonal treatment’ based on existing evidence and clinical experience. This research provides important evidence in relation to current clinical guidelines and provides a useful framework for future policy development. Further research is needed in relation to endometriosis and related conditions to ensure that decision-making is informed by robust estimates of the costs and benefits associated with different treatment options.

## Supporting information

S1 Table Embase search filter for economic studies. Search filters for economic studies designed for Embase 1974 to present.
(DOCX)

S2 Table Embase search filter for systematic reviews. Search filters for systematic reviews designed for Embase 1974 to present.
(DOCX)

S3 Table Medline search filter for economic studies. Search filters for economic studies designed for Medline1946 to present.
(DOCX)

S4 Table Medline search filter for systematic reviews. Search filters for systematic reviews designed for Medline 1946 to present.
(DOCX)

S5 Table Eligibility criteria. Inclusion and exclusion criteria used in the systematic review
(DOCX)

S6 Table Search strategy in Medline. Searches in Ovid Medline were conducted from 1946 to 29^th^ June 2016. ^**a**^Results if publications dates cover 2000–2016.
(DOCX)

S7 Table Search strategy in Embase. Searches in Embase were conducted from 1974 to 29^th^ June 2016. ^a^Results if publications dates cover 2000–2016.
(DOCX)

S8 Table Searches in Centre for Review and Dissemination (CRD) database. Publications included from year 2000 to 21^st^ of June 2016
(DOCX)

S9 Table Hand searches. (DOCX)

S10 Table Categorisation: Stage I and II. (DOCX)

S11 Table Economic evaluations. (DOCX)

S12 Table Cost studies. (DOCX)

S13 Table Utility studies. (DOCX)

S14 Table Other studies. (DOCX)

## Abbreviations

DAM: (Decision Analytical Modelling)
GP: (General Practitioner)
NICE: (National Institute for Health and Care Excellence)
NRS: (Numerical Rating Scale)
OCs: (Oral Contraceptives)
PICO: (Population, Intervention, Comparison and Outcome)
PSA: (Probabilistic Sensitivity Analysis)
QALY: (Quality Adjusted Life Year)
