# Supplementary material for: The cost-effectiveness of oral contraceptives compared to ‘no hormonal treatment’ for endometriosis-related pain: An economic evaluation
Source: PLoS One. 2019 Jan 30;14(1):e0210089. doi: 10.1371/journal.pone.0210089 (PMC6353094; doi:10.1371/journal.pone.0210089)
Supplement: S1 Table — Search filters for economic studies designed for Embase 1974 to present. (DOCX) [file pone.0210089.s001.docx]

**Table S1. Embase search filter for economic studies.**

| # | Searches | Results |
| --- | --- | --- |
| 1 | Socioeconomics/ | 121309 |
| 2 | Cost benefit analysis/ | 72069 |
| 3 | Cost effectiveness analysis/ | 114948 |
| 4 | Cost of illness/ | 16418 |
| 5 | Cost control/ | 55729 |
| 6 | Economic aspect/ | 109451 |
| 7 | Financial management/ | 107035 |
| 8 | Health care cost/ | 150713 |
| 9 | Health care financing/ | 12064 |
| 10 | Health economics/ | 35576 |
| 11 | Hospital cost/ | 16402 |
| 12 | (fiscal or financial or finance or funding).tw. | 130918 |
| 13 | Cost minimization analysis/ | 3923 |
| 14 | (cost adj estimate$).mp. | 2453 |
| 15 | (cost adj variable$).mp. | 181 |
| 16 | (unit adj cost$).mp. | 3149 |
| 17 | OR/1-16 | 755699 |
| 18 | #17 AND endometriosis.ti. | 11 |
