# Supplementary material for: The cost-effectiveness of oral contraceptives compared to ‘no hormonal treatment’ for endometriosis-related pain: An economic evaluation
Source: PLoS One. 2019 Jan 30;14(1):e0210089. doi: 10.1371/journal.pone.0210089 (PMC6353094; doi:10.1371/journal.pone.0210089)
Supplement: S2 Table — Search filters for systematic reviews designed for Embase 1974 to present. (DOCX) [file pone.0210089.s002.docx]

**Table S2. Embase search filter for systematic reviews.**

| # | Searches | Results |
| --- | --- | --- |
| 1 | exp meta analysis/ | 110203 |
| 2 | ((meta adj analy$) or metaanalys$).tw. | 121853 |
| 3 | (systematic adj (review$1 or overview$1)).tw. | 101089 |
| 4 | or/1-3 | 215904 |
| 5 | cancerlit.ab. | 677 |
| 6 | cochrane.ab. | 54300 |
| 7 | embase.ab. | 54824 |
| 8 | (psychlit or psyclit).ab. | 963 |
| 9 | (psychinfo or psycinfo).ab. | 12622 |
| 10 | (cinahl or cinhal).ab. | 16491 |
| 11 | bids.ab. | 519 |
| 12 | (fiscal or financial or finance or funding).tw. | 130918 |
| 13 | or/5-12 | 214790 |
| 14 | reference lists.ab | 12812 |
| 15 | bibliograph$.ab. | 20972 |
| 16 | hand-search$.ab. | 5724 |
| 17 | manual search$.ab. | 3516 |
| 18 | relevant journals.ab. | 1021 |
| 19 | or/14-18 | 40030 |
| 20 | data extraction.ab. | 15411 |
| 21 | selection criteria.ab. | 25023 |
| 22 | 20 or 21 | 38936 |
| 23 | review.pt. | 2167960 |
| 24 | 22 and 23 | 18579 |
| 25 | letter.pt. | 940670 |
| 26 | editorial.pt. | 509951 |
| 27 | animal/ | 1766501 |
| 28 | human/ | 17268470 |
| 29 | 27 not (27 and 28) | 1325441 |
| 30 | or/25-26,29 | 2760351 |
| 31 | 4 or 13 or 19 or 24 | 388084 |
| 32 | 31 not 30 | 37330 |
| 33 | Endometriosis.ti. AND 32 | 427 |
