# Supplementary material for: The cost-effectiveness of oral contraceptives compared to ‘no hormonal treatment’ for endometriosis-related pain: An economic evaluation
Source: PLoS One. 2019 Jan 30;14(1):e0210089. doi: 10.1371/journal.pone.0210089 (PMC6353094; doi:10.1371/journal.pone.0210089)
Supplement: S3 Table — Search filters for economic studies designed for Medline1946 to present. (DOCX) [file pone.0210089.s003.docx]

**Table S3. Medline search filter for economic studies.**

| # | Searches | Results |
| --- | --- | --- |
| 1 | Economics/ | 26727 |
| 2 | "costs and cost analysis"/ | 44254 |
| 3 | Cost allocation/ | 1989 |
| 4 | Cost-benefit analysis/ | 66406 |
| 5 | Cost control/ | 20919 |
| 6 | Cost savings/ | 9844 |
| 7 | Cost of illness/ | 20694 |
| 8 | Cost sharing/ | 2122 |
| 9 | "deductibles and coinsurance"/ | 1534 |
| 10 | Medical savings accounts/ | 499 |
| 11 | Health care costs/ | 31107 |
| 12 | Direct service costs/ | 1100 |
| 13 | Drug costs/ | 13347 |
| 14 | Employer health costs/ | 1082 |
| 15 | Hospital costs/ | 8906 |
| 16 | Health expenditures/ | 15432 |
| 17 | Capital expenditures/ | 1971 |
| 18 | Value of life/ | 1021 |
| 19 | Exp economics, hospital/ | 5503 |
| 20 | Exp economics, medical/ | 21569 |
| 21 | Economics, nursing/ | 13890 |
| 22 | Economics, pharmaceutical/ | 3937 |
| 23 | Exp "fees and charges"/ | 2623 |
| 24 | Exp budgets/ | 28327 |
| 25 | (low adj cost).mp. | 12863 |
| 26 | (high adj cost).mp. | 25237 |
| 27 | (health?care adj cost$).mp. | 8511 |
| 28 | (fiscal or funding or financial or finance).tw. | 89853 |
| 29 | (cost adj estimate$).mp. | 1549 |
| 30 | (cost adj variable).mp. | 33 |
| 31 | (unit adj cost$).mp. | 1674 |
| 32 | (economic$ or pharmacoeconomic$ or price$ or pricing).tw. | 185251 |
| 33 | Or/1-32 | 502016 |
| 34 | Endometriosis.ti. AND 33 | 140 |
