# Supplementary material for: The cost-effectiveness of oral contraceptives compared to ‘no hormonal treatment’ for endometriosis-related pain: An economic evaluation
Source: PLoS One. 2019 Jan 30;14(1):e0210089. doi: 10.1371/journal.pone.0210089 (PMC6353094; doi:10.1371/journal.pone.0210089)
Supplement: S4 Table — Search filters for systematic reviews designed for Medline 1946 to present. (DOCX) [file pone.0210089.s004.docx]

**Table S4. Medline search filter for systematic reviews.**

| # | Searches | Results |
| --- | --- | --- |
| 1 | Meta-Analysis as Topic/ | 15026 |
| 2 | meta analy$.tw. | 77296 |
| 3 | metaanaly$.tw. | 1470 |
| 4 | Meta-Analysis/ | 67006 |
| 5 | (systematic adj (review$1 or overview$1)).tw. | 66269 |
| 6 | exp Review Literature as Topic/ | 8693 |
| 7 | or/1-6 | 146663 |
| 8 | cochrane.ab. | 36956 |
| 9 | embase.ab. | 37060 |
| 10 | (psychlit or psyclit).ab. | 859 |
| 11 | (psychinfo or psycinfo).ab. | 8793 |
| 12 | (cinahl or cinhal).ab. | 12293 |
| 13 | science citation index.ab. | 2178 |
| 14 | bids.ab. | 365 |
| 15 | cancerlit.ab. | 583 |
| 16 | or/8-15 | 57930 |
| 17 | reference list$.ab. | 11043 |
| 18 | bibliograph$.ab. | 12073 |
| 19 | hand-search$.ab. | 4234 |
| 20 | relevant journals.ab. | 805 |
| 21 | manual search$.ab. | 2580 |
| 22 | or/17-21 | 27544 |
| 23 | selection criteria.ab. | 21687 |
| 24 | data extraction.ab. | 10932 |
| 25 | 23 or 24 | 30855 |
| 26 | Review/ | 2066174 |
| 27 | 25 and 26 | 22050 |
| 28 | Comment/ | 630793 |
| 29 | Letter/ | 890362 |
| 30 | Editorial/ | 382202 |
| 31 | animal/ | 5892907 |
| 32 | human/ | 16068653 |
| 33 | 31 not (31 and 32) | 4228314 |
| 34 | or/28-30,33 | 5576585 |
| 35 | 7 or 16 or 22 or 27 | 175271 |
| 36 | 35 not 34 | 164405 |
| 37 | Endometriosis.ti. AND 36 | 213 |
