# Supplementary material for: The cost-effectiveness of oral contraceptives compared to ‘no hormonal treatment’ for endometriosis-related pain: An economic evaluation
Source: PLoS One. 2019 Jan 30;14(1):e0210089. doi: 10.1371/journal.pone.0210089 (PMC6353094; doi:10.1371/journal.pone.0210089)
Supplement: S5 Table — Inclusion and exclusion criteria used in the systematic review (DOCX) [file pone.0210089.s005.docx]

**Table S5. Eligibility criteria.**

| Criteria | Include | Exclude |
| --- | --- | --- |
| Population | - Studies enrolling any number of females affected by endometriosis | - Publications without a clear description of the analysed population - Populations that are entirely female adolescent |
| Intervention | - Studies concerned with medical therapy as treatment, or in relations to surgery, or as a factor in economic evaluations. | - Studies only concerned with endometriosis-related surgery without any attention to medical therapy |
| Comparator | - All medical therapies - Including medical device therapies | - Alternative treatments that cannot be considered as medical, for example acupuncture |
| Outcomes | - Studies describing costs, health related quality of life and economic evaluations concerning medical therapy of endometriosis - Severity of pain related to endometriosis - Epidemiology data and related parameters to inform an early stage disease model | - Studies using patient reported outcomes to assess things other than health related quality of life and pain severity of individuals affected by endometriosis-related pain, including but not limited to:   - assessments of patient personality   - endometriosis coping strategies |
| Study design | - Systematic reviews, clinical trials, meta-analyses, observational studies, economic evaluations poster presentations | - Publications without a clear description of the study design |
| Countries | - Europe, US, Canada and Australia - Countries included by Health Technology Assessment international and International Society and Pharmacoeconomics and Outcomes Research | - No restrictions |
| Language | - English | - Other languages |
| Date of publication | - Year 2000-2016 | - Studies published before 2000 |
