# Supplementary material for: The cost-effectiveness of oral contraceptives compared to ‘no hormonal treatment’ for endometriosis-related pain: An economic evaluation
Source: PLoS One. 2019 Jan 30;14(1):e0210089. doi: 10.1371/journal.pone.0210089 (PMC6353094; doi:10.1371/journal.pone.0210089)
Supplement: S6 Table — Searches in Ovid Medline were conducted from 1946 to 29th June 2016. aResults if publications dates cover 2000–2016. (DOCX) [file pone.0210089.s006.docx]

**Table S6. Search strategy in Medline®.**

| **#** | **Searches** | **Results** |
| --- | --- | --- |
| **Endometriosis, pain and medical therapy** | | |
| 1 | Endometriosis.mp. OR exp endometriosis/ | 21436 |
| 2 | Dysmenorrhea.mp. OR exp Dysmenorrhea/ | 4795 |
| 3 | Dyspareunia.mp. OR exp Dyspareunia/ | 3386 |
| 4 | (pelvi$ adj2 pain$).tw. | 6720 |
| 5 | #1 OR #2 OR #3 OR #4 | 32503 |
| 6 | “medical therap$”.tw. | 22125 |
| 7 | “oral contracepti$”.tw. | 23146 |
| 8 | Danazol.tw. | 2227 |
| 9 | Gonadotrophin.tw. | 10850 |
| 10 | (AntiTNF OR (Anti adj TNF)).tw. | 7061 |
| 11 | (progestagen$ OR progestogen$).tw. | 6687 |
| 12 | (aromatase adj inhibitor$).tw. | 5299 |
| 13 | #6 OR #7 OR #8 OR #9 OR #10 OR #11 OR #12 | 74977 |
| 14 | #5 AND #13 | 2257 |
| 15 | “randomized controlled trial”.pt. | 421614 |
| 16 | “clinical trial”.mp. OR *Clinical trial/ | 612239 |
| 17 | randomi?ed controlled trial$.tw. | 93635 |
| 18 | RCT.tw. | 10107 |
| 19 | #15 OR #16 OR #17 OR #18 | 834434 |
| 20 | exp animals/ not humans.sh. | 4268124 |
| 21 | case study/ | 1806771 |
| 22 | case report.tw. | 204098 |
| 23 | Abstract report/ or letter/ | 892541 |
| 24 | #20 OR #21 OR #22 OR #23 | 6737650 |
| 25 | #19 NOT #24 | 798761 |
| 26 | #14 AND #25 | 386 |
| **Economic evaluations** | | |
| 27 | (“economic evaluation” OR “economic model*”).tw. | 7300 |
| 28 | (“cost benefit analysis” OR “benefit adj cost*” or “cost-benefit data”).tw. | 2722 |
| 29 | (“cost effectiveness” OR “cost* adj effective*” OR “cost-effectiveness data” OR “cost-effectiveness model*”).tw. | 37434 |
| 30 | (“Cost-utility” OR Costutility OR “utility adj cost*”).tw. | 2725 |
| 31 | *Endometriosis/ec [Economics] | 19 |
| 32 | (“cost* adj disease” OR “cost data” OR “budget impact” OR “Societal perspective” OR “health service perspective” OR “burden of disease” OR “economic burden”).tw. | 14218 |
| 33 | (“indirect cost*” OR “direct cost*” OR “non-medical cost*” OR “medical cost*” OR “societal cost*” OR “patient cost*”) | 14455 |
| 34 | #27 OR #28 OR #29 OR #30 OR #31 OR #32 OR #33 | 65252 |
| 35 | #5 AND #34 | 105 |
| **Quality of life** | | |
| 36 | (“quality of life” OR QoL OR “health utilities index” OR HUI OR QALY OR “quality adjusted life years”).m_titl. | 44221 |
| 37 | (“Patient reported outcome*” OR HRQoL OR HRQL OR “EQ-5D” OR EQ5D OR EuroQol).m_titl. | 2662 |
| 38 | #36 OR #37 | 46427 |
| 39 | #5 AND #38 | 190 |
| 40 | #26 OR #35 OR #39 | 670(440^a^) |
