# Supplementary material for: The cost-effectiveness of oral contraceptives compared to ‘no hormonal treatment’ for endometriosis-related pain: An economic evaluation
Source: PLoS One. 2019 Jan 30;14(1):e0210089. doi: 10.1371/journal.pone.0210089 (PMC6353094; doi:10.1371/journal.pone.0210089)
Supplement: S7 Table — Searches in Embase were conducted from 1974 to 29th June 2016. aResults if publications dates cover 2000–2016. (DOCX) [file pone.0210089.s007.docx]

**Table S7. Search strategy in Embase®.**

| **#** | **Searches** | **Results** |
| --- | --- | --- |
| **Endometriosis, pain and medical therapy** | | |
| 1 | Endometriosis.mp. OR exp endometriosis/ | 32635 |
| 2 | Dysmenorrhea.mp. OR exp Dysmenorrhea/ | 10574 |
| 3 | Dyspareunia.mp. OR exp Dyspareunia/ | 8569 |
| 4 | (pelvi$ adj2 pain$).tw. | 11662 |
| 5 | #1 OR #2 OR #3 OR #4 | 54756 |
| 6 | “medical therap$”.tw. | 35909 |
| 7 | “oral contracepti$”.tw. | 27784 |
| 8 | Danazol.tw. | 3022 |
| 9 | Gonadotrophin.tw. | 12894 |
| 10 | (AntiTNF OR (Anti adj TNF)).tw. | 15377 |
| 11 | (progestagen$ OR progestogen$).tw. | 8062 |
| 12 | (aromatase adj inhibitor$).tw. | 8755 |
| 13 | #6 OR #7 OR #8 OR #9 OR #10 OR #11 OR #12 | 108939 |
| 14 | #5 AND #13 | 3555 |
| 15 | randomi?ed controlled trial$.tw. | 138819 |
| 16 | randomized controlled trial/ | 410685 |
| 17 | clinical trial/ or “clinical trial”.mp. | 1112025 |
| 18 | RCT.tw. | 20841 |
| 19 | #15 OR #16 OR #17 OR #18 | 1273400 |
| 20 | case study/ | 39048 |
| 21 | case report.tw. | 318476 |
| 22 | Abstract report/ or letter/ | 983491 |
| 23 | #20 OR #21 OR #22 | 1333892 |
| 24 | #19 NOT #23 | 1240035 |
| 25 | #14 AND #25 | 832 |
| **Economic evaluations** | | |
| 26 | (“economic evaluation” OR “economic model*”).tw. | 11734 |
| 27 | (“cost benefit analysis” OR “benefit adj cost*” or “cost-benefit data”).tw. | 3949 |
| 28 | (“cost effectiveness” OR “cost* adj effective*” OR “cost-effectiveness data” OR “cost-effectiveness model*”).tw. | 59204 |
| 29 | (“Cost-utility” OR Costutility OR “utility adj cost*”).tw. | 4830 |
| 30 | (“cost* adj disease” OR “cost data” OR “budget impact” OR “Societal perspective” OR “health service perspective” OR “burden of disease”).tw. | 17416 |
| 31 | (“indirect cost*” OR “direct cost*” OR “non-medical cost*” OR “medical cost*” OR “societal cost*” OR “patient cost*”).tw. | 25008 |
| 32 | #27 OR #28 OR #29 OR #30 OR #31 OR #32 | 100019 |
| 33 | #5 AND #32 | 190 |
| **Quality of life** | | |
| 34 | (“quality of life” OR QoL OR “health utilities index” OR HUI).m_titl. | 71828 |
| 35 | (“Patient reported outcome*” OR HRQoL OR HRQL OR “EQ-5D” OR EQ5D OR EuroQol).m_titl. | 5914 |
| 36 | #34 OR #35 | 76510 |
| 37 | #5 AND #36 | 436 |
| 38 | #25 OR #33 OR #37 | 1435 (1219^a^) |
