# Supplementary material for: The cost-effectiveness of oral contraceptives compared to ‘no hormonal treatment’ for endometriosis-related pain: An economic evaluation
Source: PLoS One. 2019 Jan 30;14(1):e0210089. doi: 10.1371/journal.pone.0210089 (PMC6353094; doi:10.1371/journal.pone.0210089)
Supplement: S8 Table — Publications included from year 2000 to 21st of June 2016 (DOCX) [file pone.0210089.s008.docx]

**Table S8. Searches in Centre for Review and Dissemination (CRD) database.**

| # | Searches | Results |
| --- | --- | --- |
| **DARE** | | |
| 1 | Endometriosis (Any field) | 90 |
| 2 | #1 AND pain (Title) | 16 |
| **NHS EED** | | |
| 1 | Endometriosis (Any field) | 11 |
| **HTA 2016** | | |
| 1 | Endometriosis (Any field) | 19 |
