# Supplementary material for: The cost-effectiveness of oral contraceptives compared to ‘no hormonal treatment’ for endometriosis-related pain: An economic evaluation
Source: PLoS One. 2019 Jan 30;14(1):e0210089. doi: 10.1371/journal.pone.0210089 (PMC6353094; doi:10.1371/journal.pone.0210089)
Supplement: S9 Table — (DOCX) [file pone.0210089.s009.docx]

**Table S9. Hand searches.**

| # | The following resources were hand searched for relevant material and the selection was based on clinical inputs and relevance | Results |
| --- | --- | --- |
| **Endometriosis** | | |
| 1 | international society for pharmacoeconomics and outcomes research, health technology assessment international, 3^rd^ European congress on Endometriosis Budapest, 2^nd^ congress of the society of endometriosis and uterine disorders Singapore, 13^th^ world congress on endometriosis Vancouver, European society of human reproduction and embryology, national institute for health and care excellence, Scottish medicines consortium and reference lists) | 2 |
