# Supplementary material for: The cost-effectiveness of oral contraceptives compared to ‘no hormonal treatment’ for endometriosis-related pain: An economic evaluation
Source: PLoS One. 2019 Jan 30;14(1):e0210089. doi: 10.1371/journal.pone.0210089 (PMC6353094; doi:10.1371/journal.pone.0210089)
Supplement: S10 Table — (DOCX) [file pone.0210089.s010.docx]

**Table S10. Categorisation: Stage I and II.**

| *Stage I* |
| --- |
| 1. A study that reports primary data on costs or health care utilisation, and includes a formal economic evaluation. |
| 1. A study that discuss economics aspects using primary or secondary data, or examines costs or utility data. |
| 1. A study with useful information that may inform a conceptual cost-effectiveness model allowing the comparison of medical therapies treating endometriosis-related pain, that does not fall into category (A) and (B). |
| 1. A study that does not fall into the categories (A), (B) and (C) |
| Studies coded (A), (B) and (C) were considered relevant and were taken forward for further categorisation. Those coded (D) were excluded. |
| *Stage II* |
| 1. A cost-utility analysis. |
| 1. Cost-minimisation, cost-effectiveness and cost-benefit analyses. |
| 1. A study on costs. |
| 1. A study on utilities. |
| 1. Other relevant studies. |
| 1. Studies not relevant to (1), (2), (3), (4) and (5). |
| Studies coded A(1), A(2), B(1), B(2), B(3), B(4) and C(5) were included as part of the literature searches. All studies coded (6) were rejected. |
