# Supplementary material for: The cost-effectiveness of oral contraceptives compared to ‘no hormonal treatment’ for endometriosis-related pain: An economic evaluation
Source: PLoS One. 2019 Jan 30;14(1):e0210089. doi: 10.1371/journal.pone.0210089 (PMC6353094; doi:10.1371/journal.pone.0210089)
Supplement: S11 Table — (DOCX) [file pone.0210089.s011.docx]

**Table S11. Economic evaluations.**

| Studies | Final classification | Quality assessment | Primary focus | Country | Perspective | Outcome | Modelling | Data sources |
| --- | --- | --- | --- | --- | --- | --- | --- | --- |
| (Arakawa et al., 2015) | B(1) | Moderate | Self-care vs. oral contraceptive and/or Dienogest | Japan | Societal, health service and patient perspective | Yen/QALY | Markov model | Primary / secondary data sources |
| (Araujo et al., 2009) | B(2) | Low | Goserelin therapy | Brazil | Health service | Time until reduction of symptoms / US$ | N/A | Primary / secondary data sources |
| (Lukac et al., 2011a) | B(1) | Low | Dienogest vs. GnRHa | Slovakia | Health service | € Euro/QALY | N/A | Secondary |
| (Lukac et al., 2011b) | B(1) | Low | Dienogest vs. GnRHa | Slovakia | Health service | Euro/QALY | N/A | Secondary |
| (Marmarali et al., 2012) | B(1) | Low | Dienogest vs. leuprolide and goserelin acetate | Turkey | Health service | Cost/QALY by CE plane. No ICER given. | Markov model | N/A |
| (Sanghera et al., 2016) | B(1) | High | No treatment, levonorgestrel-releasing hormone, depot-medroxyprogesterone acetate and oral contraceptive pill | UK | Health service | £ Pound sterling/QALY | State transition Markov model | Secondary |
