# Supplementary material for: The cost-effectiveness of oral contraceptives compared to ‘no hormonal treatment’ for endometriosis-related pain: An economic evaluation
Source: PLoS One. 2019 Jan 30;14(1):e0210089. doi: 10.1371/journal.pone.0210089 (PMC6353094; doi:10.1371/journal.pone.0210089)
Supplement: S12 Table — (DOCX) [file pone.0210089.s012.docx]

**Table S12. Cost studies.**

| Studies | Final classification | Quality assessment | Primary focus | Country | Data sources | Currency | Year of currency | Comment |
| --- | --- | --- | --- | --- | --- | --- | --- | --- |
| (Bostrom et al., 2012) | B(3) | Low | Endometriosis and effect on direct/indirect costs | Sweden | Primary | SEK/Euro | N/A | ISPOR PIH14: Presentation with primary data. Limited extent of detail is given. |
| (D'Hooghe et al., 2011) | B(3) | Moderate | Endometriosis associated costs | International | Primary | Euro | N/A | P-215: Costs estimation of health service costs associated with endometriosis and QALY estimation are given. |
| (Fuldeore et al., 2015) | B(3) | High | Endometriosis costs, before and after diagnosis | United States | Primary | $ USD | Inflated to 2010 | First study to estimate cost prior to diagnosis. Costs are high around diagnosis, before and after. Population adults: Adolescent and post-menopausal women excluded due to cost variation. |
| (Gao et al., 2006a) | B(3) | Moderate | Overall economic burden of endometriosis | United States | Secondary | $ USD | Not inflated to current year, but referenced | Systematic review with societal perspective on indirect and direct costs associated with endometriosis. Unlikely to inform modelling, but contains many useful references. |
| (Oppelt et al., 2012) | B(3) | Moderate | Inpatient costs of endometriosis | Germany | Primary | € Euro | 2006 | Mostly collect inpatient cost data, where a substantial amount of the procedures is surgery. Medical therapy inpatient costs cannot be deducted from this study. |
| (Prast et al., 2013) | B(3) | High | Direct and indirect costs | Austria | Primary | € Euro | 2009 | Study with medium sample size, but very detailed in the reporting. Especially table 3, with prevalence and costs per type of medication can prove useful in modelling. |
| (Simoens et al., 2007) | B(3) | High | Review of direct and indirect costs | International, but extrapolated to US | Secondary | $ US Dollars | 2002 | Useful review covering the direct medical costs of medical therapy. Interesting discussion of costs between surgery and medical therapy. |
| (Simoens et al., 2011a) | B(3) | High | Study protocol | International | Primary | € Euro | 2009 | Study protocol to raise awareness. Table 1 is useful and covers the most important cost drivers in terms of medication, diagnosis and health care providers. |
| (Soliman et al., 2016) | B(3) | High | Review | International | Secondary | $ US | 2013 | Review covering many cost with a useful table for comparison of studies. |
