# Supplementary material for: The cost-effectiveness of oral contraceptives compared to ‘no hormonal treatment’ for endometriosis-related pain: An economic evaluation
Source: PLoS One. 2019 Jan 30;14(1):e0210089. doi: 10.1371/journal.pone.0210089 (PMC6353094; doi:10.1371/journal.pone.0210089)
Supplement: S13 Table — (DOCX) [file pone.0210089.s013.docx]

**Table S13. Utility studies.**

| Studies | Final classification | Quality assessment | Primary focus | Country | Data sources | Outcome measure | Comment |
| --- | --- | --- | --- | --- | --- | --- | --- |
| (Brandes et al., 2010) | B(4) | Moderate | Quality of life | International | Primary | Quality of life (SF-36) | Recruiting favoured patients with severe endometriosis. Quality of life had negative correlation with duration of disease. |
| (Caruso et al., 2015) | B(4) | Moderate | Quality of life | Italian | Primary | Quality of life (SF-36) | Figure 3: Contains SF-36 scores for baseline, 3 months and 6 months’ treatment with dienogest. |
| (Di Donato et al., 2013) | B(4) | Moderate | Quality of life and sexual function | Italian | Primary | Quality of life (SF-36) and sexual function (SHOW-Q) | Identifies dyspareunia, dysmenorrhea, dyschezia and chronic pelvic pain are associated with reduction in QoL. Qol scores decreased with number of previous surgeries. |
| (Gao et al., 2006b) | B(4) | High | Quality of life | International | Secondary | Many different utility measures | Systematic review covering Health related quality of life instruments by surgery and medical therapy. Good overview of different approaches used and their comparisons. |
| (Jia et al., 2012) | B(4) | High | Quality of life | International (English language) | Secondary | Endometriosis influence on health related quality of life | Focus is more on treatments, their impact endometriosis and health related quality of life. The strengths of evidence of each comparison are rated from strong to weak, or inconclusive. |
| (Jones et al., 2006) | B(4) | High | Validation of Quality of life questionnaire | United Kingdom | Primary | Endometriosis Health profile-30 | Aimed to carry out secondary factor analysis, rests of reliability and validity for the endometriosis health profile-30. |
| (Lovkvist et al., 2012) | B(4) | Low | Quality of life | Sweden | Primary | Short form 36 (SF-36) | M066: Quality of life survey by SF-36 in women with endometriosis shows lower scores than general population. A poster presentation. |
| (Lovkvist et al., 2016) | B(4) | High | Age-related quality of life | Sweden | Primary | Questionnaire including SF-36 | Mean age first physician visit and at diagnosis reported. Results show decrease in QoL with age, opposite general population. Indicates that age-related QoL should be accounted for in a model. |
| (Miller, 2000) | B(4) | High | Pain and quality of life | Illinois | Primary | SF-36 and endometriosis symptom severity scale | Clinical trial that follows patients for the first four weeks of the gonadotropin stimulatory phase. Shows temporary decrease in QoL and a placebo effect. |
| (Muneyyirci-Delale et al., 2014) | B(4) | Low | Quality of life | N/A | Primary | Endometriosis health profile-30 | Leuprolide acetate depot vs Norethindrone acetate. Improves QoL, but no significant changes between the two. |
| (Nunes et al., 2012) | B(4) | Low | Quality of life | N/A | Primary | SF-36 questionnaire | Quality of life in women suffering from endometriosis is worse than in women without. |
| (Porpora et al., 2013) | B(4) | Low | Quality of life | Rome | Primary | WHOQOL and Italian MFSQ | Pain was a major patient concern and the authors suggest a psychological approach together with pain management. |
| (Souza et al., 2011) | B(4) | Moderate | Quality of life and Visual analogue scale (VAS) | Brazil | Primary | VAS and WHOQOL-BREF | Diagnosed CPP produces same QoL as mild endometriosis. Significant reduction in QoL if patients are classified as High CPP. |
| (Tekin et al., 2012) | B(4) | Low | VAS and verbal rating scale (VRS) | Turkey | Primary | WHOQOL-BREF, VAS and VRS | Poster presentation that concludes that dysmenorrhea is related to lower QoL, but CPP, dyspareunia might not. Not relevant to modelling. |
| (Unsal et al., 2010a) | B(4) | High | HRQoL | Turkey | Primary | VAS and SF-36 | Only concerned with dysmenorrhea, but table VII has an elaborate list comparing severity stages with SF-36. Ideal for modelling, but scores need conversion to QALYs. |
