# Supplementary material for: The cost-effectiveness of oral contraceptives compared to ‘no hormonal treatment’ for endometriosis-related pain: An economic evaluation
Source: PLoS One. 2019 Jan 30;14(1):e0210089. doi: 10.1371/journal.pone.0210089 (PMC6353094; doi:10.1371/journal.pone.0210089)
Supplement: S14 Table — (DOCX) [file pone.0210089.s014.docx]

**Table S14. Other studies.**

| Studies | Final classifcation | Study type | Year | Country | Primary focus | Comments |
| --- | --- | --- | --- | --- | --- | --- |
| (Dunselman et al., 2013) | C(5) | Guideline | 2013 | International | Management of endometriosis for empirical, medical, surgical, pre- or postoperative medical treatment | A comprehensive guideline that classifies recommendations by the evidence strength. These management strategies for different types of pain and their medical treatments can be used to inform and define modelling stages. |
| (Schroder et al., 2004) | C(5) | Systematic review of treatments | 2004 | International | The paper covers different treatment and describe future treatment. | Describes different treatments, but the description of future treatments is obviously not updated due to the publication date. |
| (Simoens et al., 2014) | C(5) | Poster presentation | 2008 | International | A study that collects both costs and quality of life data | Health care costs per woman €3113, but with quality of life as the best predictor of direct health care costs. Endometriosis-associated symptoms generated 0,809 quality-adjusted life years. |
| (Simoens et al., 2011b) | C(5) | Study | 2012 | International | A study that collects both costs and quality of life data | Equivalent to (Simoens et al., 2014). More elaborate data on QALY including std. errors and costs. Can inform some input parameters in modelling. |
| (NICE, 2014b) | C(5) | Guidance | Expected 2017 | United kingdom | NICE guidance on endometriosis: diagnosis and management | As the project is not finished, scoping, consultations, stakeholder list and comments are available. Informative in terms of considerations that are made together with the comments. |
